# Integrating Health and Social Care Services

**DOI:** 10.1177/2164957X221117112

**Published:** 2022-08-05

**Authors:** David Blockley, Gordon Stirrat, Kirsty Alexander, Sabrina Phillips

**Affiliations:** 1 Civil Engineering, 1980 University of Bristol, Bristol, UK; 2 Obstetrics & Gynaecology, 1980 University of Bristol, Bristol, UK; 3 GP & Chair Bristol North and West Locality Leadership Group at Bristol, 1976 North Somerset and South Gloucestershire CCG, Bristol, UK; 4 Lambeth Living Well Network Alliance, Lambeth, London, UK

**Keywords:** health policy, health care, integrative medicine, public health

## Abstract

**Background:**

A recent UK Government draft Heath White Paper follows the NHS England long term plan when it states that NHS England requires “a new framework that builds on changes already being made as well as building in the flexibility to support the system to tackle challenges of the future”. At present the structure of Health and Social Care Services UK reporting to Government seems unhelpfully complex and opaque.

**Objective:**

The purpose of this paper is to contribute to the building of a new framework using a generic approach to identify and use ‘systemic processes’ to facilitate the integration of Health and Social Care services in NHS England and elsewhere.

**Methods:**

We highlight some of the critical issues that are currently hindering integration and set out a new way of understanding the structure of NHS England through an ‘inside-out’ analysis of systemic processes.

**Results:**

We describe and give three examples of existing systemic processes as ‘Consulting a patient’, ‘Enhancing a Single point of access’ - to mental health services and ‘Delivering health and social care services England’.

**Conclusions:**

Rethinking the interactions between existing organisations could arguably bring considerable benefits including cost savings, better co-ordination, less ‘admin’ stress on staff where the work is done and provide more organisational adaptability in an uncertain future. Ultimately our suggestions are aimed at helping people to deliver better patient care - the impelling purpose of all health and social care services.

## Three Questions and Answers

1.What do we already know about this topic?The methodology was developed for engineering systems. We are exploring its application in health and social care.
2.How Does Your Research Contribute to the Field?It is a new approach
3.What are your research’s implications towards theory, practice, or policy?It could help to ‘join-up’ or integrate processes to deliver whole person care and hence improve efficiency and make cost savings.

## Introduction

UK Government draft Health White Papers (WP),^1,2^ follow the NHS England long term plan,^3^ which states that NHS England requires “a new framework that builds on changes already being made as well as building in the flexibility to support the system to tackle challenges of the future”. The WPs recognise that legislation is “at best, an enabler of organisations and individuals, outlining the ways…which… will encourage positive behaviours and innovations, remove barriers, enable flexibility, reduce bureaucracy, and is capable of adapting over time’. The document is a response of the UK Government to “the greatest challenge our health and care systems has ever faced” - the coronavirus pandemic - an unprecedented test of our health and care services. The extraordinary dedication, care and skill of the people who work in our communities and hospitals is a reminder of how precious our health and care services are. The draft WP recognises the real progress of our organisations collaborating with each other to provide ‘joined-up’ or integrated services for ‘whole-person’ care to enable staff to work outside of organisational silos to deliver more patient-centred and personalised approaches to care. The term ‘joined-up’ is used commonly in the UK to mean a system characterised by coordination and coherence of thought. A joined-up system is an integrated and comprehensible whole that focuses on producing purposeful consistent rational strategies, tactics, and results at many levels. All parts of the system, such as departments or sections, communicate well with each other and act together towards agreed goals efficiently and effectively. The term joined-up derives from the time of the UK prime minister Tony Blair and his first ‘New labour’ government of 1999. They saw a need for better collaboration across government departments dealing with shared issues. The commitment was set out in a UK Government White Paper ‘Modernising Government’.^4^ The aim was to ensure ‘policy making is more joined-up and strategic '. The term subsequently disappeared from front line political debate in the UK but is still frequently referred to for integrating services of various kinds. For example, the WP^1^ refers to a requirement that different parts of the NHS join up better. Joining-up is part of title of the latest NHS report^2^ where it is stated that the proposals will ensure a more joined-up approach between health and social care. Encouraging more joined-up working between hospitals and social care for services following discharge is one of the aims of^5^ which has so far failed to materialise. For more detail on building bridges between theory and practice by joined-up systems thinking in engineering see Blockley.^6^ The NHS reports^1-3^ recognise that much has yet to be done. As O’Donnell and Begg^7^ note ‘*the UK … appears to have entered the crisis with a weak evidence base – the government may have been operating without the right structures and processes in place in order for it to …make effective decisions…… a particular area of weakness seems to have been the ability of central and local governments to collaborate.*’

The heart of the new proposals is to provide joined-up integrated whole-person care for everyone in England. In doing so the role of the private sector will be reduced, and greater control taken by Government Ministers. Government is recognising that the NHS 2012 reforms have “*in some cases hindered integration between providers*” and not acted as a ‘market’ in the way originally intended. The WP implies that Government will leave the NHS and local authorities to run services but whilst encouraging them to work together more effectively. Emphasis will be placed on reducing bureaucracy and improving integration and promoting healthy behaviour and preventative health care.

## Purpose

The purpose of this paper is to develop our suggestions made previously^8^ that identifying and using ‘systemic processes’ can significantly facilitate the integration of NHS England Health and Social Care services. Specifically, our objectives are to:

1. summarize the philosophy and methodology developed in an engineering context, point out some criticisms and suggest how it is relevant to the practice of health care worldwide,
2. highlight some of the critical issues that are currently hindering integration,
3. set out a new way of understanding the structure of NHS England and the way it works through an ‘inside-out’ analysis of systemic processes,
4. describe and give examples of some NHS systemic processes,
5. outline a strategy for identifying and improving them across the entire health and care community,
6. reflect on the possible improved outcomes in health and social care systems outside of NHS England.

## Systems Thinking Through Systemic Processes

Research studies of engineering failures over a period of 30 years have shown that technically qualified professionals often do not adequately address issues at the interface between technically complex problems (for example innovative structural forms such as box girder bridges in the 1970s) and human and organisational difficulties (for example political, economic, and social pressures that cause cost overruns, delays, and inefficiencies). In a series of papers^6,9-19^ the investigations led to an approach that aims at reducing the gaps between what we know, what we do and why things go wrong. The approach is based on systems thinking. Systems thinking, in summary is ‘joined-up’ thinking. It is rooted in Aristotle’s seven circumstances of *who, what, where, when*, together with *in what way,* and *by what* [efficient] *means (how)* from his Nicomachean Ethics.^19^ It concerns getting the right information (what) to the right people (who) in the right form (context – where) in the right way (how) for the right reason (why) at the right time (when). A central concept in systems thinking is that of a holon as first suggested by Koestler.^20^ A holon is both a whole and a part *at the same time*. A whole is made up of parts. A set of parts interact to create a whole and these interactions create emergent properties of the whole that are not found in the parts. For example, both you and I are holons. As individuals we are wholes that have the emergent properties that we can walk and talk (plus many other attributes that contribute to making us who we are). Our parts are our subsystems (musculoskeletal, nervous, circulatory, respiratory, digestive, hormonal, excretory and reproductive) that cannot by themselves walk and talk. But they are, in turn, also holons with properties that emerge from the interactions between organs. Again, our organs are holons with properties that emerge from interactions between cells. We humans are also parts (of our family, of various social, and political groups and organisations). Again, these groups are holons as individual groups which are part of wider groups – for example local councils are parts of regional councils which are part of state and federal governance. Ultimately, we are part of the entire human race, which is part of the world and the universe. Humanity is part of nature, and this should never be forgotten. Indeed, by this thinking every object or *thing*, whether natural or manufactured is a holon - whether a seed, flower, tree, valley, or mountain and whether a bridge, building, car, aeroplane, computer, or transport system. Holons fall naturally into levels with the entire universe and the top and the smallest sub-atomic particle or event at the bottom – that is called a holarchy. Every layer of the holarchy is important but we choose to model a set of *layers* according to our purpose in building that model. The reasons why thinking about systems in terms of holons is important is, at minimum, 6-fold. First, it makes us focus on the purpose of any modelling we undertake. Second, it enables us more easily to model with high dimensionality. Third, context becomes clearly apparent and important. Fourth, relationships both vertically (by logical entailment) and horizontally (by connectivity) are easier to identify and model.^21^ Fifth, the attributes of a holon are self-similar (at a certain level of abstraction) which provides us with a certain level of simplicity out of complexity. Finally, uncertainty in relationship between holons become easier to identify. Although not discussed here, the capturing of uncertainty as impredicativity as set out by Blockley et al^21^ is a much more general and practical concept than probability. Impredictivity is the gap between the rules of syntax of a language and the semantic meaning. It enables us to think about vagueness and incompleteness of practical decision making and action more realistically. In brief, thinking with holons provides practical clarity to our modeling of systems.

The task is to identify each holon as a connected systemic process. The connections vertically form the layers or levels of the entire system – lower levels being logically entailed by higher ones. The connections horizontally interact to create emergent properties which include the changing extent to which the processes are ‘on track’. That extent, at any point in time, is assessed by a measure of the progress towards the purpose of the process. Importantly the measure maybe uncertain and incomplete with evidence for and against and possibly in conflict. Formally, the uncertainty can be captured as an interval probability using an Italian Flag as a colourful measure of the evidence.^17,22^ In this way incubating problems, as Turner and Pidgeon^23^ showed in studies of many man-made disasters across many types of systems, may be identified. Turner and Pidgeon showed that as difficulties over time interact and grow, they may begin to inhibit or threaten progress, possibly cause damage, and eventually, if the signs are unheeded, become ‘an accident waiting to happen’ and a final trigger event causes total failure of the system. By identifying and monitoring systemic processes those responsible can heed the warning signs and ameliorate the problems before they become too serious. For more detail see Ref. 17,18,23.

In this paper we explore the use of these ideas for health care. We identify NHS England as a complex holon analogous to a living organism. The part holons cascade from the very top (in England the Minister for Health and Social Security) through the medical and administrative staff right down to the humble cleaner of a hospital ward. All involved have tasks to perform which, we suggest, can be captured as systemic processes. If these processes are not properly ‘joined-up’ and the system is not well integrated, then changes can be made by those responsible to connect the processes. In many hospitals individual procedures such as treating cataracts or inserting ICD implants are well documented but often in different forms whilst data bases and appointments systems are not. The identification of systemic processes, in a kind of gigantic jigsaw of holons, may make it easier for those, with the responsibility, to join them up.

## Issues That Hinder Integration

In a previous report^8^ we have highlighted that the NHS is complex set of organizations which, for the most part, work quite well despite a myriad of sub systems. We noted that other authors have also referred to this complexity^24-27^ and, for example Powell^26^ says “The leadership of the NHS seems fractured……a system under siege where success isn’t celebrated but failure is catastrophised”. We need “to build the confidence of the people who hold the problem.” Attempts to explain the complex systems are necessarily partial.^27-30^ Good people can make a poor system work well but when pressures overwhelm them failure can be catastrophic.^31^

In^8^ we asked what are the incentives that will make integration work? Why should the many providers take responsibility for the total care of the whole population for probably less resource – when they have very little control over all the parts?

The pandemic has demonstrated to all that everyone in the NHS shares a strong common purpose of ‘caring for our patients’. But does that translate into more detailed shared purposes at the various levels of the sub-systems? We see at least 7 groups of issues that currently hinder integration.

1. *Resources - w*here adequate resources do not follow activity without waste through unforeseen issues. For example, the cost of drugs from commercial pharmacies (and thus to the NHS overall) is considerably greater than the same drugs from hospital pharmacies Hospitals may initiate medication for a patient for which they have a particular financial deal, but the primary carers are not part of the deal, and the medication may become very expensive for them. When payment models do not encourage closer working around the needs of patients by sharing ‘pain and gain’ in an agreed manner then integration will be harder to achieve.
2. *Organisations -* the various parties to Integrated Care have to be aware that their potential partners may be fearful of extra work and responsibility being ‘dumped’ onto them. Sometimes, for example, individual GPs may be reluctant to hold challenging risks that hospital staff, with their more collective responsibility, may not be exposed to. If a patient dies during hospital treatment after a late diagnosis of bowel cancer caused at least partly through unintended delays (in the systems and perhaps also by the behaviour of the patient) the GP may find himself/herself in the spotlight of blame by relatives, for not diagnosing quickly enough. Co-operating organisations in the public and private sectors may have different bureaucratic constraints and work to different priorities and time scales as they attempt to adjust with agility to rapid changes. Organisational culture (in simple terms, the way that things are done – including the unwritten rules that influence behaviour and attitudes) can often dominate strategic aims. Some of the factors that influence culture include leadership, deployment of resources, clarity of structure and processes, values and traditions. Success rests on leadership that overcomes professional silos and tribalism with good IT and access to targeted data – to harmonise strategy and culture.
3. *Social care –* where the relationships and interactions within and between health and social care need to be improved. Much depends on funding sources. Health care as maintaining/improving the physical and mental well-being of people (whether children, young people, adults, or the elderly) by preventing, diagnosing, treating, or curing of disease, illness, injury, and other physical and mental impairments, is distinct from but intimately related to social care. In the UK primary health care is provided by a general practitioner. Secondary care is from specialists whilst tertiary care is even more highly specialized. Social care, on the other hand, is personal care. Usually this is given by public or private organisations to help people who need specialised assistance to live a comfortable, healthy, and fulfilling life as far as is possible. One acute example of a lack of integration in the UK is so called ‘bed blocking’. In 2021 there was an estimated 10,000 thousand patients^32^ occupying hospital beds waiting delayed discharge until the next stage of their care becomes available. Delays may be lack of temporary or permanent space in a residential home, or rehabilitation unit, or a smaller community hospital, or a lack of a supportive care package for their return home. In 2022 the BMA^33^ reported longer waits for patients due to the build-up of significant backlogs after Covid-19. Saltman and Figueras^34^ identified four broad themes that influence organization and behaviour within nearly all western health care systems and these, if not clearly articulated, can hinder integration. They are the role of state and private sector market, the level of decentralisation, the rights of patients and the role of public health promotion, disease prevention and lifestyle choices. The distinction between Europe the USA is an emphasis on collective responsibility vs individual and financial accountability. Papanicolas et al^35^ conceptualize health care as organized action with four dimensions: maintaining values and meaning, producing by integrating and stabilizing, adapting to acquire resources, and achieving goals. They conclude for example that responses to benchmarking comparisons can become defensive through a need to justify policy rather than the constructive process of searching for new ideas and ways of working. Shengelia et al^36^ suggest a probability measure of effective coverage of health gain from an intervention. They list the six issues that hinder integration as; true need may not be perceived need because of differences in cultural norms; specific and overall interventions may not cover cross-cutting systemic barriers; high-quality interventions may not lead to the right diagnosis; inadequate assessment of demand and supply; lack of a framework for effective coverage of the individual as well as the population; and finally, an insufficient ex ante view of effective coverage.^36^ A review of the health and social care systems of 9 countries^37^ concluded that major reforms take time and depend on local context with no one country or model of provision emerging as ideal. NHS England is unique in its low level of cost sharing. All of the other eight charge user fees with many having private health insurance. Some countries have mandatory insurance to cover social care costs, but many do not function well although the gap between health and social care is less stark than in England. Very few countries have insurance schemes that cover accommodation and daily living costs in residential care even though they make up a large proportion of the costs to residents. The role of the family differs between countries and means testing can create perverse incentives such as choosing types of care based on finance rather than need. In Korea and Sweden long-term care insurance schemes are administered by the national health insurance programme. In Germany health insurance funds administer the long-term care insurance programmes and most people get both types of insurance from the same provider. The United States was the one example among the countries profiled of more integrated funding of health and social care, as the Medicaid programme provides safety net coverage of both health and some social care for Americans with very low incomes. Japanese health care providers are expanding into social care provision to integrate delivery in one organisation. The Netherlands is moving from social insurance model for health care to managed competition between private insurers. The United States has had sweeping reforms of the private health insurance market with government subsidies to expand coverage. France, Germany, Japan, and Korea have introduced social insurance schemes to pay for long-term care; and in Ireland the movement is currently underway towards a Dutch-style system of health care financing.
4. *Targets* that are set top down without adequate consultation can lead to perverse behaviour. For example, waiting list targets resulted in requests for investigations and letters of referral being hidden from the doctors so that the patient was not put on an already “over target” waiting list. During the pandemic urgent cases were delayed because some doctors were not permitted to see the requests. Doctors and other staff were being told to use particular “pathways” and follow “guidelines”. Because the hospitals will not defend the staff if they stray from the guidelines then they effectively become directives. This leads to ossification of thought and procedure.
5. *Leadership* - top-down control of the NHS is seen by many to be inefficient and has hindered much of its work - particularly during the pandemic. Nevertheless, it can be effective when the main task is relatively simple, for example immunisation (once all the vaccine available). However, diagnosis and treatment processes for individual patients are not simple – they are beyond the abilities of top-down management to control. Leadership cannot come from managers who do not understand or who are not qualified to do the job. The old system of consultant firms in the hospitals worked because the person in charge knew what they were doing. Likewise, the sisters on the wards and the matron in the hospital were a good combination of knowledge and experience. During the pandemic doctors in the NHS were told not to use various drugs that they might have wished to repurpose – such as ivermectin, hydroxychloroquine and dexamethasone. Until double blind trials had been undertaken these drugs were forbidden from use. This was seen by many as an unhelpful interruption of clinical freedom with consequent harm to patients.
6. *Feedback and Learning - a* consequence of top-down control is that *s*taff are not encouraged to put their ideas forward. Some live-in-fear of their jobs if they *blow the whistle* to anybody other than their line manager – the very person that they often wish to report. Organisational learning requires more freedom of expression. As Arora et al report^38^ innovation is key, but support pathways are fragmented with notable redundancies. They stress the importance of digital innovations and interoperability (joined-upness) of data systems.
7. *Integrating at all levels* - there is little evidence of an equivalent attention to ‘joining-up’ the proliferation of higher level non-local and fragmented^39^ organisations reporting to or sponsored by the DHSC. The drive for integration ‘where the work is actually done’ does not appear to be reflected at the top of the NHS. There seems to be no unifying concept around which the integration can coalesce nationally. Without that there is a significant risk of regional ad hoc solutions that may not join-up across geographical/national boundaries.

## Systemic Processes

In Ref. 8 we suggested that the entirety of the health and social care system may be better understood by identifying its existing *‘systemic processes’*. The purpose would be to evolve a more ‘joined-up’, less fractured and more integrated ‘whole’ across regional boundaries.

A systemic process^17,22^ is not a sequence of events as in a flow chart as defined by NHS Improvement Guides^40,41^ - although the practical advice for running workshops is useful. Neither it is simply a series of actions towards an end. Rather it is a reconceptualization of process as a potential that drives a flow of change – just as the volts of a battery drive an electrical current or water pressure drives the flow of water. A systemic process captures why and what people actually do and how change happens.

The primary innovative characteristic of systemic processes is that they are structurally self-similar – just like the pieces of a jigsaw. Process ‘pieces’ form clusters and clusters of clusters which we can think of as layers of parts of the whole. The processes are, of course unlike a jigsaw, dynamic and ever changing. Systemic ‘jigsaw’ processes capture that change. Change derives from a potential that drives a flow (or dually from flow that creates potential). In classical physical systems potential is electromagnetic or gravitational and flow is movement. For example, voltage, current and velocity, force. In human affairs the potential is contained in answers to questions ‘why’ – purpose, aims and objectives. The change is contained in answers to questions ‘who, what, where, when’. The transformation of the flow from one ‘state of affairs’ to another is contained in answers to questions ‘how’. The aim is to model the right information ‘what’ (data as performance indicators, success criteria and shared care records), for the right reasons ‘why’ (purpose), to the right person or organisation ‘who’ (role, stakeholder), in the right way ‘where’ (context) and at the right time ‘when’.

Systemic processes are wholes and parts at the same time. They are ‘being things’ that change through natural forces – living or inanimate. You and I are ‘being’ wholes as individuals and yet also parts of family and social groups. As individuals ‘wholes’ we are made of parts such as our muscular skeleton structure and digestive systems. We are as we are because the parts collaborate to form the whole – in other words we show ‘emergent’ characteristics.^42^ We see the NHS as an ecology of interdependent relations and interactions the behaviour and characteristics of which *emerge* from interactions between systemic processes - just as a whole jigsaw picture *emerges* from the interlocking pieces of a jigsaw

The successful delivery of a systemic process derives from the success of sets of processes in the adjacent layers. An important part of the identification of lower sub-system processes is that their successes are jointly necessary and sufficient for the process above – there is an explicit logical relationship connecting defined success, in all of its manifestations, at every level. It is also important to note that the layers are not hierarchical power structures. Rather they are levels of abstraction from setting policy down to detailed implementation. The attributes of each systemic process can be grouped under the headings of why, how, who, what, where and when. At each level the attributes can be identified (by multiple players) in a common format. These can then be implemented on a secure intranet to be accessed by those given authority to do so. Every process should have one ‘process owner’ responsible for leading the players involved in that process to detect and monitor progress, to identify potential unintended consequences and agree the required actions to steer the process to success. Success is defined as meeting purpose/aims/objectives and avoiding failure.

Practically we find it helpful to name a systemic process using the present participle or ‘ing’ form. For example, ‘Doing something’ – ‘Testing a blood sample’ or ‘Diagnosing a condition’. During the ‘piecing together’ or the ‘building of clusters’ systemic processes, changes and improvements will suggest themselves. For example, points of strategic dissonance may become apparent. This happens when an organisation hangs on to the old ways of doing things for too long because of a disconnect between actions and intent or purpose at any given level. For more on the details of this approach see Ref. 17,22

## A Possible Strategy

In Ref. 8 we suggest that the key to driving change should be ‘influencing from the bottom up’. Top-down centralisation has proven ineffective.^1^ Ham^43^ writes ‘The overcentralised management of the pandemic was undoubtedly a factor in the failure to learn more effectively. Boris Johnson, the UK prime minister, and a small number of Cabinet members were visible in their leadership and appeared reluctant to draw on the expertise and intelligence of the devolved administrations, regional, and local government leaders. Opportunities for learning were lost, contributing to the mistakes that were made’. Policy decisions for change have to make sense to those ‘on the front line’ if they are to respond positively. That response is also conditional on the need for change to evolve up and down through the entire system. Given that policy makers and staff need to see how their decisions spread or percolate through the organisation we now propose that rather than describing the change process as ‘bottom-up’ we should regard it as being ‘inside-out’. By that we mean that change should be initiated at *all levels* of the system. The first stage of that change process is to identify the systemic processes at that level. Then by discussing the overlaps and interactions with ‘neighbouring’ systemic processes the people involved (led by the process owners) can adjust or adapt the systemic processes to fit. It is as if we are reshaping our jigsaw pieces so that they fit and interlock. These kinds of adapting change processes would not be ‘one-off’ and static. Rather they are dynamic systemic *learning* processes and an integral part of an ongoing improvement model. Organisational adaptability through feedback and learning is a key to success as successful change is shared and spreads out from many different points across the clusters to the whole organisation.

A strategy such as this will require high level policy makers and those allocating resources to also adapt and integrate. That can be difficult for political leaders who may be criticised by media and public alike for changing their policies. There are two solutions to this dilemma. One is to declare that running the NHS is no longer a political matter. The other is to take the opportunity given to us by the pandemic to initiate a different approach to openness to change when faced with a future full of uncertainty – with many unknown unknowns. This would require considerable political skill to persuade everyone in an accountable democratic society that certainty is simply no longer available in complex systems. Changing one’s mind is currently seen as a weakness and not, as it should be, a strength when fully justified by dependable evidence. Leaders will need to learn and adapt to facilitate the changes that will incentivise all those affected by their decisions to work differently. The strategy has also to apply to the totality of health and social care services of the organisations in and related to NHS England, including local authority social care and private companies.

In summary we suggest that the strategy to implement the laudable aims set out in the WP^1^ should be to understand and motivate - not by imposing top-down targets but by ‘inside-out’ growing of clusters of understanding with commensurate motivation to ‘join up processes at multiple points and levels whether at the top, middle or on the front line.

## Three Examples

We noted in Ref. 8 that the organisations reporting to the DHSC seem to be contributing to seven high level systemic processes. They are Commissioning Care, Providing Care, Supplying, Regulating, Advising, Educating and Researching. They will form our third example – see below. First, we present two lower level examples with a little detail (but still incomplete for space reasons) for illustration.

**Figure 1.**
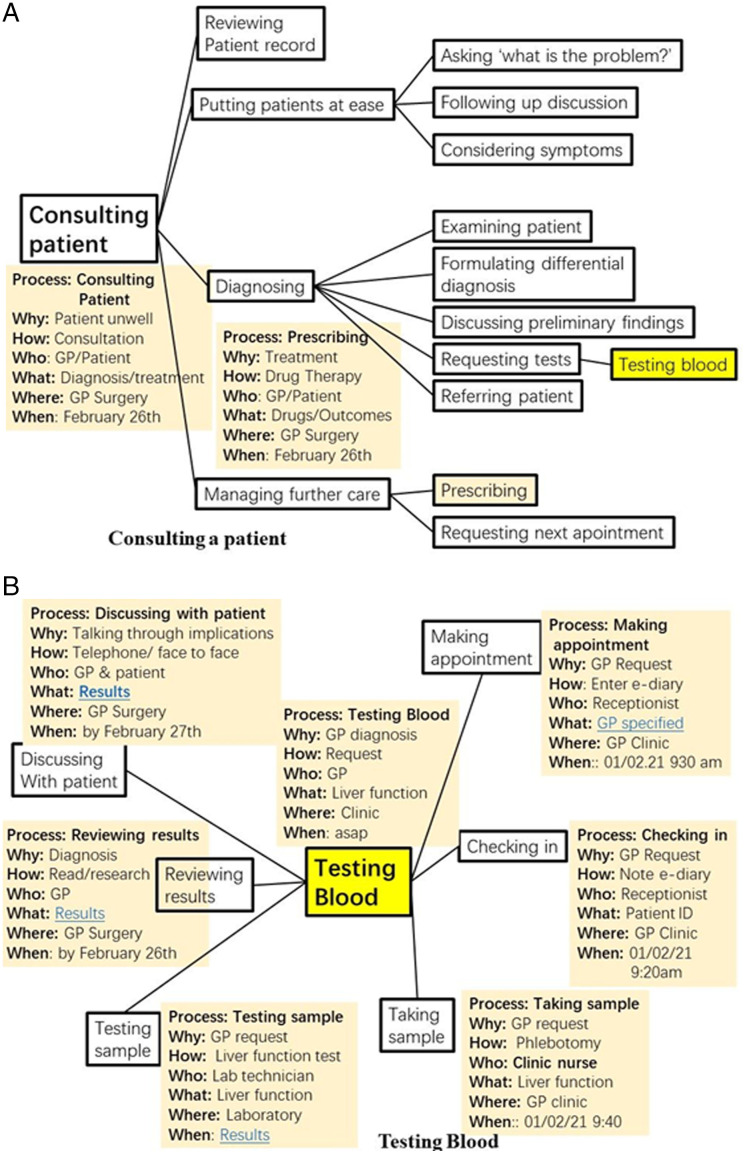
(A) Consulting a patient. (B) Testing blood.

The method (a how question/attribute) we use to identify systemic processes begins with setting down, as a Mind Map^44^ what people actually do. Figure 1A represents the systemic process of a GP ‘Consulting with a Patient’. Necessarily, as we have said, the diagram is incomplete. However, the main sub-processes shown are ‘Reviewing the patient record’, ‘Putting patient at ease’, ‘Diagnosing’ and ‘Managing further care’. The test for deciding on the nature of the sub-processes is to ask the question: ‘Would the successes of the sub-processes be jointly necessary and sufficient for the success of the process? Of course, each of these sub-processes has sub-sub-processes as shown. However, the sub -processes are self-similar in structure – they are each a whole and a part at the same time – which is why they are holons.^20^ A holon sub-process of ‘Diagnosing’ is ‘Requesting tests’ and a holon sub-process of that in turn is ‘Testing blood’ as highlighted in yellow. Each systemic process holon has a set of attributes gathered under the headings of why, how, who, what, where and when. In Figure 1A these are given in an abbreviated form. In a fully implemented Mind Map the attribute descriptions could be fuller and hold hypertext links to documents held on an intranet. For example, the (who) patient attribute could link to a Share Care Record or similar patient file. The (who) GP attribute could link to a GP Practice website of that GP. A link from the why attribute could link to present or previous symptoms. The Figure cannot show all attributes for all processes even as abbreviated. A further example shown in the diagram is that of ‘Prescribing’ with possible attributes also as shown. Figure 1B is a continuation of Figure 1A for the systemic process of ‘Testing Blood’. Subprocesses of ‘Making an appointment’, ‘Checking in’ etc. are shown again with abbreviated attributes. Of course, these processes are familiar and normally well managed by GPs which is why we have given them as illustrative examples that relate to something familar.

**Figure 2.**
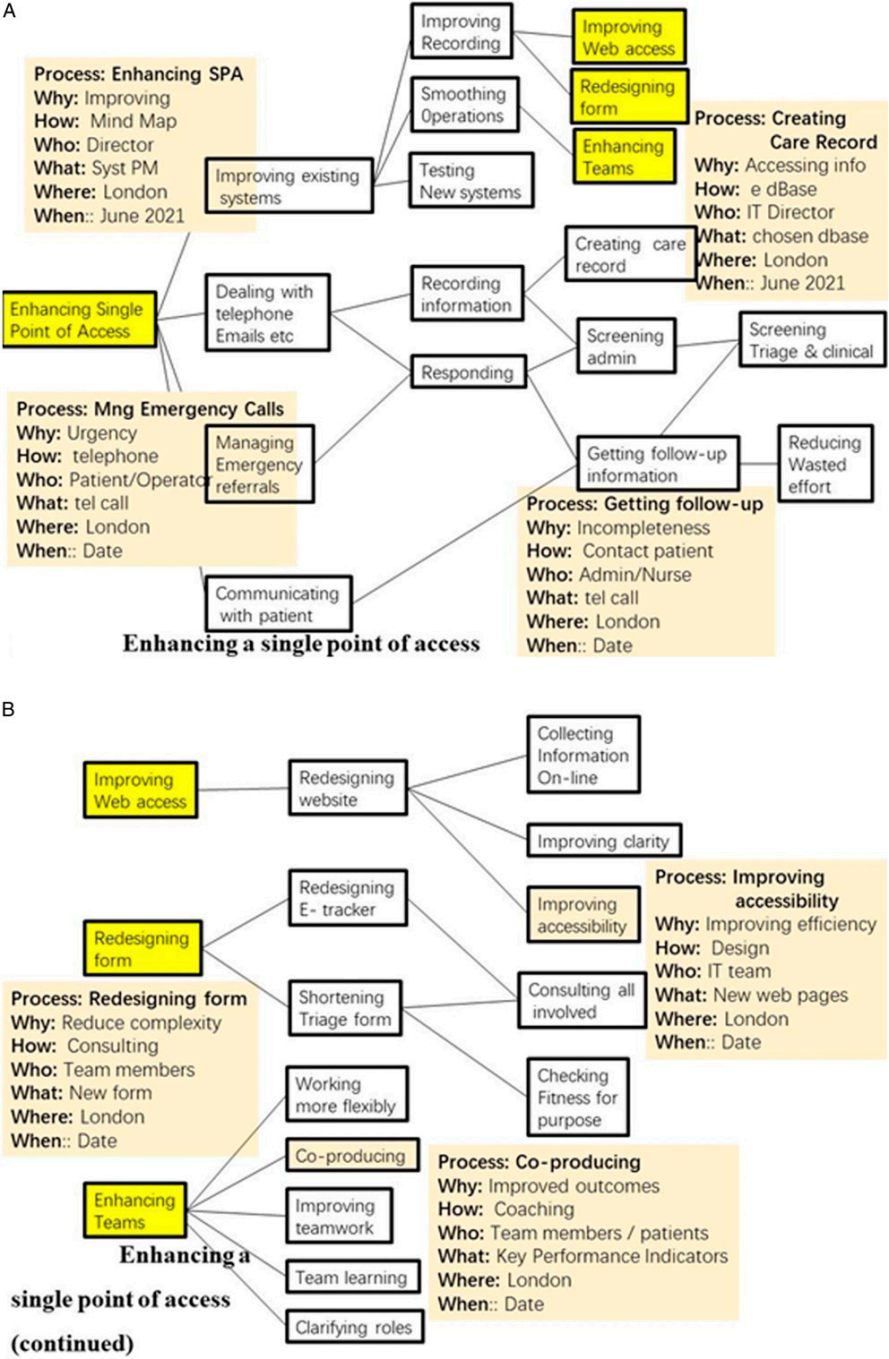
(A & B) Enhancing a single point of access.

Figure 2A is less familiar and more challenging. It shows an ongoing adjustment of systemic processes in the integrated Alliance Network of the fourth author. The process is ‘Enabling a Single Point of Access’ for mental health patients. Currently there are multiple points of access and these need to be integrated.

The processes highlighted in yellow of ‘Improving web access’ and Redesigning forms’ and Enhancing Teams are shown in Figure 2B. Clearly there are more processes and attributes to identify – for example ‘Consulting all involved’ and Checking fitness for purpose. Again a full description is not possible in this paper and work is ongoing.

**Figure 3.**
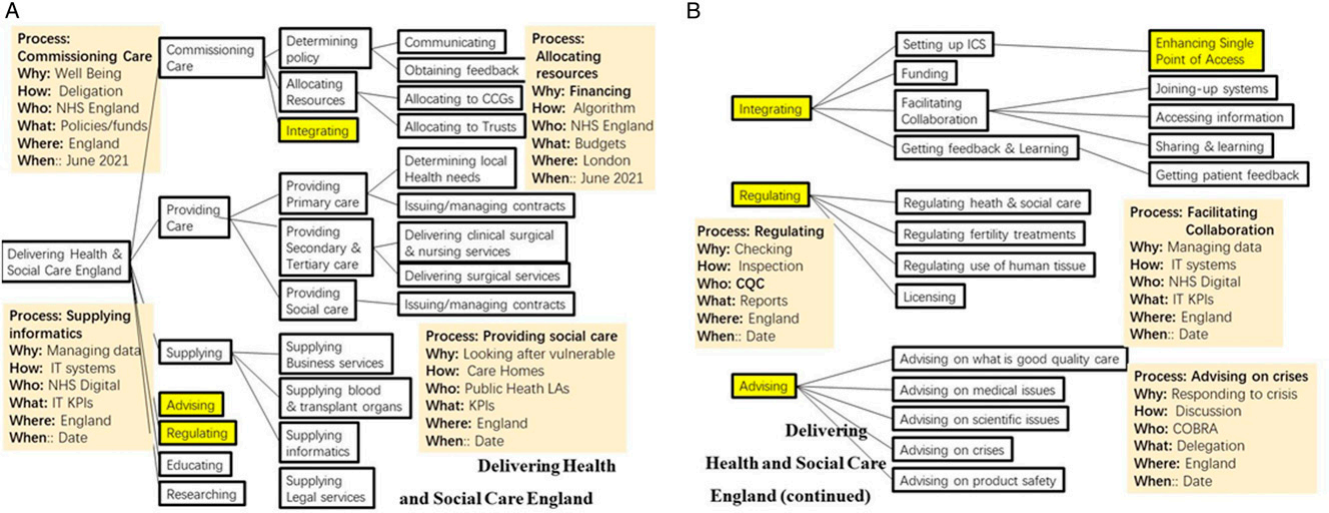
(A & B) Delivering health and social care England.

A third example shown in Figure 3A is even more challenging. It is the top-level process of ‘Delivering Health and Social Care England’ and has the 7 sub-processes identified at the head of this section and in 8. Sub-processes included are ‘Determining policy, ‘Allocating Resources’ and Integrating’ (highlighted in yellow). These are continued in Figure 3B together with ‘Regulating’, and ‘Advising’. Again, in each case we identify the attributes shown in abbreviated form for only some of the processes.

Clearly it is impossible here to capture the full richness of the processes and their attributes. That task can be made viable through adequate software operated by multiple players who ‘own’ each individual systemic process on multiple servers connected by an intranet. In the first instance only a simple mind Mapping tool is required but a full implementation will require adequate software with built in permissions and safeguards.

## Relevance to International Health Care Services

The discussion so far has largely related to NHS England, but the ideas and methodologies presented are generic and may be applied to any health care system. As stated earlier the ideas were developed for engineering applications by the first author but the other three authors, all of whom have practiced or are currently practicing in NHS England are trialling the potential for healthcare systems – indeed Example 2 is ongoing, and the results will be reported in due course. As Gold^45^ remarks *‘Looking at different countries’ healthcare systems is like looking at the NHS in a circus mirror: the main elements are all there, but it’s a different image. There are differences between almost all countries’ organisational structures, along with the way their ‘insurance’ system is administered, usually for historical reasons. And while European citizens can use other countries’ public healthcare under the European health insurance card scheme, attempts to join systems up (such as patient records) are at an early stage*.’

The Commonwealth Fund^46^ has compared 20 health systems across the world including Australia, Brazil, Canada, China through to the USA. The review includes the role of government, public and private health insurance, services covered, primary care, administrative arrangements, after hours care, hospitals, mental and long-term care, digital records, integration, and coordination. As expected, there are similarities and differences, but all are striving to improve data coordination and information systems. By using a generic system for attaching data to systemic processes based on why, how, who what, where and when, then a better more unified performance can be achieved through integrated joined-up processes. The ideas and methodologies are generic because they:

1. are independent of the content or attributes of the processes owned and modelled by the professional participants themselves;
2. apply to all types of arrangements of heath care regardless of local differences;
3. can be adapted to any culture or context;
4. have been developed out of an intention to progress towards quality as fitness for purpose and to enable identifying incubating issues (early warning signs) and hence to avoid failure – especially where complex technical processes (such as tricky medical procedures meet complex organisational processes (such as ensuring adequate funding);
5. highlight needs, adequacy, and effectiveness (as why – criteria and what – progress and performance questions), access (as where questions). It enables the players (who attributes) acting as team leaders (process owners) or as team players to address questions of diversity on the basis of ethnicity, culture, gender, age, and others as found to be relevant locally.

In this way all relevant aspects are covered by the methodology. The quality of that cover depends on the modeller or modelling team. The important point is that the people who do the work are empowered to make localised changes to processes in liaison with other affected processes. Where issues cannot be resolved locally suggestions can be fed up through the holarchy. The methodology is independent of state or private funding. It allows a type of decentralisation which can be harmonious with high level decision making if the culture is appropriate. It facilitates the rights of patients and the promotion of public health through the defining of suitable processes It is stressed that the attributes are not treated as simply technical or tactical but can be used to address any entailed complexities – indeed the methodology is intended to address those complexities head on by identifying any incubating problems as described earlier. These will include issues such as the perpetuation of epistemic injustices, status, safety, and working conditions of staff, that can be best addressed by those who are most familiar with them and not ‘brushed under the carpet’. All the hindering factors highlighted earlier can be addressed by those practitioners most affected and where required transmitted through the holarchy to ‘higher-level’ overall processes. For example, true local processes can be designed to manage differences in cultural norms and cross-cutting systemic barriers. Checks on diagnosis and interventions can be incorporated. Assessments of demand and supply and effective coverage through suitably designed processes as can ex ante analyses.^36^ Likewise, those most affected by bed-blocking are empowered to incorporate changes in the processes for which they are responsible and then to liaise with the other ‘horizontal’ processes in the holarchy that seem to be causing the delays. Ultimately if the issues are financial or higher level systemic (such as providing more nursing care homes) then again by feeding back up through the holarchy suggestions for change by those trying to operate the systems could have greater credence.

## Criticisms of the Methodology

A major criticism of the systemic process methodology is that it requires a big change of ‘mindset’ for those involved. A particular difficulty is that the traditional and widely accepted separation of ‘product’ from ‘process’ are brought together and combined into one concept. Products are not merely ‘things’ but processes. A simple ‘table’ is modelled as a process since it has a life cycle that evolves and changes in time. All data are integrated, understood, and managed as the attributes of systemic processes. A table has a purpose of ‘being a table’ (why), has been conceived, designed, and made (how) and has attributes such as dimensions, and materials (what), set in a context or place (an operating theatre) for a time (age). Likewise patient records are a dynamic process with a life cycle of constant change and not a simple static file of information. We have found from experience that this can be hard for some to accept. Data are important but are not the primary focus. The focus is on purpose. Data are contextual attributes of processes and have to be interpreted locally by those qualified to do so.

Consequently, training in the ideas and ways of ‘systems thinking’ is not straightforward and so success may well be ‘patchy’. Other major difficulties are an acceptance of holons as both a whole and part, an ability to define adequate boundaries between holons as processes so that physical and social systems are related through what people do and how they perform. Awareness of context and the need for qualified interpretation is of crucial importance and so often neglected in traditional thinking. For example, it must be made clear when the adopted definitions move out of context and hence are no longer appropriate. These issues are directly related to how the purpose of a systemic process is defined. In some cases, that purpose is straightforward but in others it may well be problematic – especially for higher level management/social processes. Collaborative, cooperative teamwork will be essential in resolving any issues and that may require a change of culture. The implied hierarchy of holons must be understood not as a power structure but rather one of abstraction of levels of purpose. The whole purpose of the methodology is to empower those most affected to design and meet their own purposes and objectives in harmony with the many constraints imposed by being a part of a very large organisation. At the same time keeping track, with accountability and transparency, of all major performance attributes including progress towards stated purpose and objectives. The extent to which these criticisms are successfully addressed is a matter of the effectiveness of the modelling and the need to change and evolve the systems processes that created them.

## Improving Outcomes

In summary the approach described could:

1. provide an overview of the whole organisational structure as a purpose driven ecology of interdependent relations and interactions between systemic processes;
2. enable everyone to understand and appreciate that structure and to trace performance and accountabilities right down from the top-level policy making to the detailed ‘caring for patient’ processes;
3. allow people to identify overlaps and interdependencies. For example, advisory organisations could possibly be streamlined to co-ordinate advice and avoid incongruity and inconsistencies;
4. enable caring for the sick, caring for public health and the vulnerable to be under one ‘umbrella’ to avoid inconsistencies of policy over the longer time scales than politicians are typically in post;
5. enable a common data structure for the why, how, who, what, where and when attributes of each systemic process – important to avoid different groups using different data structures that cannot easily be shared and for data exchange via an intranet, with appropriate permissions to sensitive data, and the monitoring of progress and interventions to steer processes towards success;
6. enable all those involved in delivering success for the NHS to identify how the necessary and sufficient conditions for the success of systemic processes where the work is actually done with feedback into success and deficiencies at higher policy levels;
7. permit practitioners to clarify
  • pathways of precise terms of delegation and accountability;
  • how decision makers at all layers of the system delegate responsibilities down through the layers of processes – for example from government to NHS England and others to workers where the work is done and ultimately to the patients;
  • how decision makers at all layers of the system are accountable up through the system – for example from hospital ward care to Trust Boards, CCGs, NHS England and to government, parliament and ultimately to the public;
  • remove, reduce or ameliorate inconsistencies between policy and practice;
  • improve adaptability of response to unintended consequences and future unknowns such as pandemics and potential impacts from climate change;
  • evolve and change the culture where required to purposeful adaptive collaboration between the various parts of the organisation.

## Conclusions

1. The structure of Health and Social Care Services UK reporting to Government seems unhelpfully complex and opaque. We suggest a rationalisation using a ‘systems thinking bottom-up’ approach would be more likely to succeed than yet another top-down reorganisation.
2. Rethinking the interactions between existing organisations around ‘systemic processes’ could arguably bring considerable benefits including cost savings, better co-ordination, less ‘admin’ stress on staff at the where the work is actually done and provide more organisational adaptability in an uncertain future for all health and social care systems.
3. Help practitioners to identify and ameliorate incubating problems that inhibit progress and could trigger total system failure.
4. Ultimately ‘systemic processes’ could help everyone deliver better patient care because that is the impelling purpose of the NHS.
